# Emerging Microfluidic Approaches for Platelet Mechanobiology and Interplay With Circulatory Systems

**DOI:** 10.3389/fcvm.2021.766513

**Published:** 2021-11-25

**Authors:** Yingqi Zhang, Savindi De Zoysa Ramasundara, Renee Ellen Preketes-tardiani, Vivian Cheng, Hongxu Lu, Lining Arnold Ju

**Affiliations:** ^1^School of Biomedical Engineering, Faculty of Engineering, The University of Sydney, Darlington, NSW, Australia; ^2^Charles Perkins Centre, The University of Sydney, Camperdown, NSW, Australia; ^3^Heart Research Institute, Newtown, NSW, Australia; ^4^School of Medicine, The University of Notre Dame Sydney, Darlinghurst, NSW, Australia; ^5^Faculty of Science, Institute for Biomedical Materials and Devices, The University of Technology Sydney, Ultimo, NSW, Australia

**Keywords:** microfluidic, platelet, mechanobiology, thrombosis, hemodynamic, endothelial cells, von Willebrand factor, COVID-19

## Abstract

Understanding how platelets can sense and respond to hemodynamic forces in disturbed blood flow and complexed vasculature is crucial to the development of more effective and safer antithrombotic therapeutics. By incorporating diverse structural and functional designs, microfluidic technologies have emerged to mimic microvascular anatomies and hemodynamic microenvironments, which open the floodgates for fascinating platelet mechanobiology investigations. The latest endothelialized microfluidics can even recapitulate the crosstalk between platelets and the circulatory system, including the vessel walls and plasma proteins such as von Willebrand factor. Hereby, we highlight these exciting microfluidic applications to platelet mechanobiology and platelet–circulatory system interplay as implicated in thrombosis. Last but not least, we discuss the need for microfluidic standardization and summarize the commercially available microfluidic platforms for researchers to obtain reproducible and consistent results in the field.

## Introduction

While three determinants for thrombosis—hypercoagulability, endothelial dysfunction, and hemodynamics—are outlined in Virchow's triad (1), platelets play an essential role in arterial thrombosis. Their thrombotic functions are governed by a range of biomechanical factors and the underlying mechanobiology is a rapidly evolving field and topical research area (2). Intriguingly, platelets respond to flow disturbance in complex vessel architectures with respect to bifurcation, branching, stenosis and aneurysm (3), which inevitably produce local shear gradients (4–6), vorticity (7) and even turbulence (4, 8). The biomechanical platelet adhesion and aggregation are primarily mediated by its two mechanoreceptors—glycoprotein Ib (GPIb) and glycoprotein IIb/IIIa (GPIIb/IIIa or integrin α_IIb_β_3_) (9, 10). Furthermore, to facilitate thrombus stabilization, platelets generate contractile forces which are mediated by cytoskeletal components (actin, tubulin, Arp2/3) and motor proteins (myosin and dynein) in response to the mechanical microenvironment (11, 12). Meanwhile, platelets interact with red blood cells, neutrophils, plasma proteins [von Willebrand factor (VWF), fibrinogen], and the vessel wall (endothelial cells) to exert their hemostatic and thrombotic functions (13). Increasingly, microfluidic technologies have emerged as powerful and indispensable approaches to investigate the mechanosensitive behaviors of platelets (2, 12, 14). Numerous endothelialized microfluidics have been invented to model the interplay between platelets, endothelium and VWF under physiologically relevant hemodynamic microenvironments (6, 15, 16). In the context of platelet mechanobiology, we summarize these state-of-the-art microfluidic methodologies that have recently been invented in the field.

## Microfluidic Approaches for Investigating Platelet Mechanobiology and Platelet–Circulatory System Interplay

Single-cell biomechanical nanotools such as atomic force microscopy (17), optical tweezers (18) and micropipette based adhesion assays (19) significantly advanced our understanding of platelet mechanobiology (20). However, a major shortcoming of these *in vitro* techniques is their inability to recapitulate the physiologically relevant hemodynamics in the context of platelet adhesion and aggregation (21). To this end, animal models of thrombosis and intravital microscopies have become more popular and accessible approaches for examining thrombus initiation, progression and propagation *in vivo* (22). Nevertheless, multiple fundamental limitations still exist with the animal approaches (23, 24): (i) inter-species variabilities which prevent complete recapitulation of human disease pathogenesis; (ii) physiological variations between individual animals; (iii) lengthy ethical approval processes.

Shear-based *in vitro* assays, such as parallel plate flow chambers (25), cone-and-plate viscometers (26) and thromboelastometers (27) have been widely used for investigations of human blood samples under physiologically relevant shear rates and shear stresses (5, 28–30). Although these methods were instrumental in the current understanding of shear dependent platelet thrombosis, to some extent, they are limited due to the need for large sample volumes, low throughput, and their inability to accurately emulate vascular architectures and mechanical properties of the circulatory system (31–33). To these points, microfluidic techniques have rapidly emerged as complementary humanized models for investigations of platelet mechanobiology and biomechanical thrombosis. More recently, the International Society of Thrombosis and Hemostasis (ISTH) have even made recommendations for producing reliable microfluidic devices with reproducible thrombogenic coating and consistent hemodynamic design (34). This represents a major step forward in promoting microfluidic technologies for future pre-clinical and diagnostic testing methods in hemostasis and thrombosis.

Moreover, while most of the research interests focus on large vessel thrombosis, there is an emerging trend toward understanding thrombosis in the microcirculation which is significantly implicated in a variety of systemic disorders such as: Thrombotic Thrombocytopenia Purpura (TTP) and Hemolytic Uremic Syndrome (HUS) which lead to multi-organ dysfunction syndrome and death (35, 36); and localized organ injuries resulting from trauma, ischemia-reperfusion, transplant rejection, disseminated intravascular coagulation, sickle cell disease and more recently COVID-19 (35, 37, 38). The microfluidic approach can effectively recapitulate the anatomical structures within such small scale as opposed to conventional shear-based assays (39). The recent advancements of microfluidic designs and the micro and nano fabrication—particularly the shift from devices with a single layer to those with multiple-layered structures (40), and from parallel straight channels to complex geometries (15)—have enabled the investigation of platelet mechanobiology in more complicated vascular biomechanical microenvironments.

In the past decade, soft lithography has rapidly grown to enable high-fidelity micropatterning. Parallel straight channels can simply be fabricated by the single-layered photolithography and poly(dimethylsiloxane) (PDMS) casting processes, resulting in a vascular geometry with laminar flow (5, 16, 41, 42). When connected with external syringe pumps, such simple microfluidic setting enables systematic investigation of shear dependent platelet adhesion (43) and aggregation (44). Additionally, vascular cell cultures can be incorporated; specifically, endothelialized microchannels can model the interaction between blood components and the vessel wall under physiological hemodynamic parameters (45), or even when subjected to proinflammatory or other endothelial diseased conditions (16).

Furthermore, single-layered microfluidics can emulate more complex vessel structures, such as bifurcation (39), 2D stenosis (46), network (47), and micropost array (40) to recapitulate flow disturbance induced platelet activation, adhesion, contraction and aggregation (48, 49). The mechanical stimuli investigated include elevated shear stress (50, 51), shear rate gradient (52–54), vorticity (7) and turbulence (8, 55) at different locations of the microchannel. Moreover, lining the microchannel with endothelial cells enables the investigation of prothrombotic synergistic effects of hemodynamic forces, blood cells and the vessel wall (56, 57). Rapid prototyping methods such as vertical milling molding (58) and 3D printing (56) enable fabrication of microfluidics with circular structures and variations in the z-direction. PDMS casting around poly(methyl methacrylate) (PMMA) optical fibers can also create circular channels (59, 60). As such, these recent microfluidic advancements provide great means for examining how shear stress, shear rate gradients, vorticity, blood viscosity and contractility affect platelet thrombotic functions.

In the past 3 years, *in vitro* lung (61), blood vessel (62), and tumor models (63) were made on microfluidics (64). These biomimetic organ-on-a-chip models recapitulate the structural, functional, and mechanical aspects of vascular microenvironments. Obviously, emulating such hierarchical organs requires emerging microfabrication methods including 3D bioprinting (65), double-layered soft lithography (66), injection molding (67), and micropattern stamping (68). This allows the production of multiple-layered structures via one-time or assembled fabrication. The composite microsystems can support tissue coculture and the channel-channel interface, thereby enabling studies of bidirectional tissue signaling across the endothelial barrier (61). More importantly, external mechanical manipulation apparatus (e.g., stiff ECM matrices, actuation chambers) can be integrated for disease-specific studies and rapid drug screening (55, 69). Although multiple-layered microfluidics incur higher fabrication requirements including precise alignment and assembly, they present physiologically relevant hemodynamic microenvironments for better biomimetic performance.

**Figure 1:**
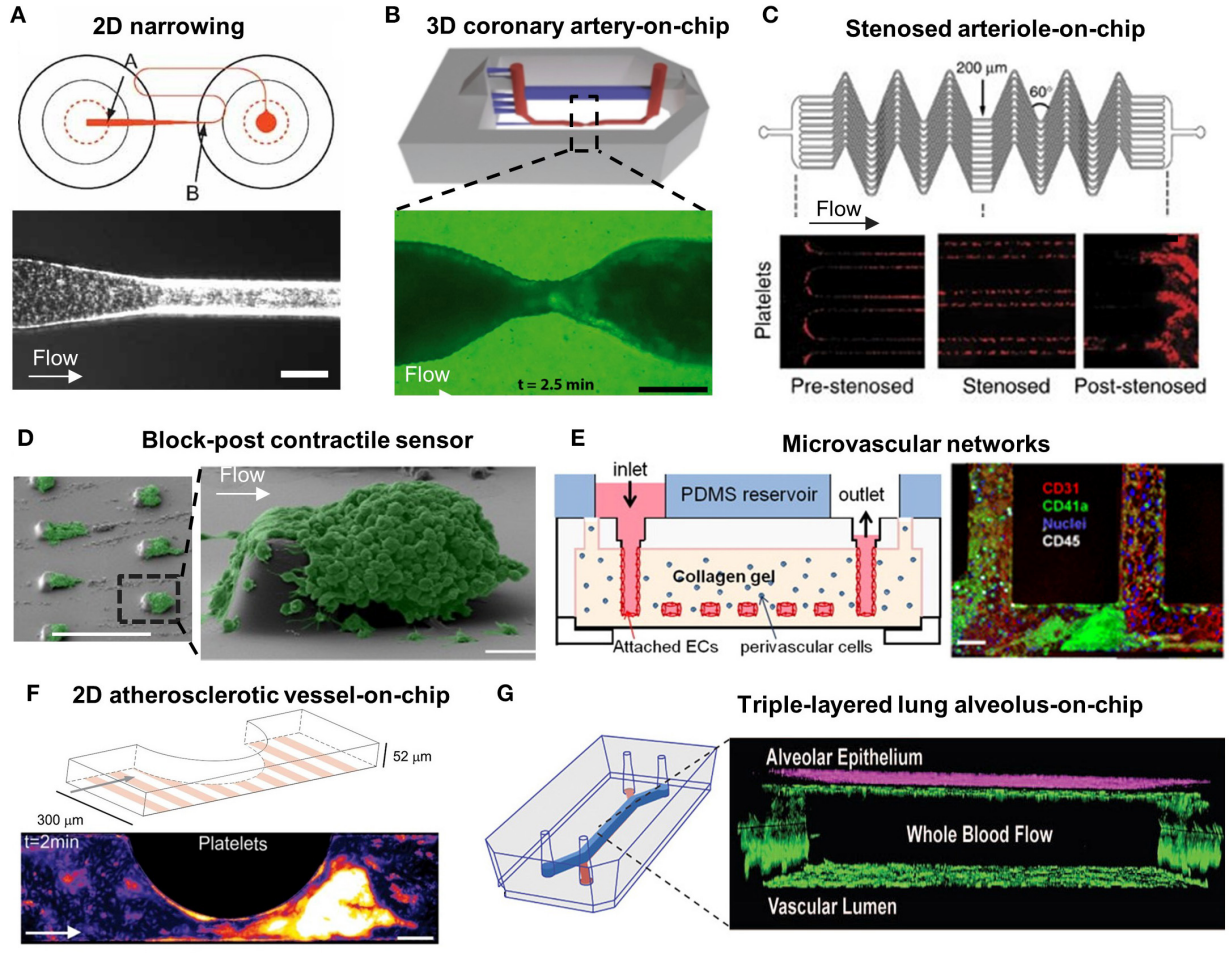
Microfluidic models for the study of platelet mechanobiology and its interplay with circulatory system in thrombus development**. (A)** A 2D Narrowing channel from Conant et al. (70); Top: A well-plate microfluidic design, where arrow A indicates microfluidic channel entrance and arrow B indicates the neckdown region; Bottom: Platelet behavior at the neckdown zone. Scale bar = 100 μm. **(B)** A 3D coronary artery-on-chip model from Costa et al. (56); Top: Animation image of the 3D microfluidic model; Bottom: Fluorescence image of platelet aggregation at stenosis. Scale bar = 200 μm. **(C)** Stenosed arteriole mimicking microfluidic device from Jain et al. (71); A network of parallel stenosed microchannels which contain multiple regions of pre-stenosis, stenosis and post-stenosis. **(D)** Block-post contractile sensor from Ting et al. (40); SEM images of platelet aggregation on a micropost microfluidic device. Scale bar = 10 μm. **(E)** Microvascular networks from Zheng et al. (67); Left: Schematic of the multiple-layered collagen network in the microfluidic device; Right: Confocal image of the endothelialized microfluidic channel with platelet (green), leucocytes (white) adhesion and aggregation. Scale bar = 50 μm. **(F)** A 2D atherosclerotic vessel-on-chip model from Westein et al. (6); Left: Schematic diagram of microfluidic device incorporating microchannels with varying degrees of stenosis (20–80%); Right: Confocal images of platelet aggregates at the endothelialized stenotic region upon blood perfusion. Scale bar = 100 μm. **(G)** Triple-layered lung alveolus-on-chip model from Jain et al. (61); Left: Schematic of the three-layered alveolus-on-a-chip model; Right: Confocal image of endothelial and epithelial cell coculture in the device.

Last but not least, the transparency of PDMS material is greatly compatible with advanced microscopies to visualize thrombotic dynamics in real time. These PDMS or equivalent microfluidics thus offer a versatile way to evaluate platelet activation, adhesion, aggregation, morphological change, and soluble agonist secretion under hemodynamic control. A range of studies have delved into reproducing diverse vessel architectures to investigate how biomechanical factors (e.g., shear stress, shear gradients and traction forces) and the circulatory components (e.g., vessel endothelium and VWF) regulate platelet functions. As shown in the Figure 1 and Table 1, we summarize these recent lab-on-chip approaches in the context of platelet mechanobiology and thrombosis.

**Table 1:** Summary of microfluidic models for platelet mechanobiology in thrombotic diseases.

**Microfluidics description**	**Fabrication method**	**Mechanical profile**	**Vasculature status**	**Perfusion sample**	**Functionality test**	**References**
**Single layered simple structure microfluidics**						
Straight channel	Soft lithography	Pump-driven constant shear input and output	HUVECs w/wo TNF-α stimulation	Recalcified human WB	Thrombus formation or platelet function assay	(16, 41, 42)
Straight channel	Soft lithography	Pump-driven constant shear input and output	Primary HMVECs	Recalcified human WB	Clot formation upon diabetic complications	(45)
**Single layered complex structures microfluidics**						
Ladder network channel	Soft lithography	Pump-driven constant flow input and geometry mediated flow output	CGN/TF coated bare channel	Recalcified human WB	Shear rate gradient-dependent platelet adhesion, aggregation	(47)
Bifurcation channel	Soft lithography	Pump-driven constant flow input and geometry mediated flow output	HUVECs/HLMVECs	Human WB	Hematologic microvascular occlusion	(118)
Junction with multiple inlets and single outlet	Soft lithography	Pump-driven constant flow input and output	HUVECs	Recalcified human WB	Outside-in interference of thrombosis	(72)
2D stenosis channel	Soft lithography	Pump-driven constant flow input and geometry mediated flow output	Non-coated bare channel	Citrated human WB	Shear rate gradient-dependent platelet adhesion, aggregation	(46)
2D stenosis channel with non-uniform height	Aluminum vertical milling molding; Soft lithography	Pressure-driven constant flow input and geometry mediated flow output	Type I CGN coated bare channel	Porcine blood	Shear-dependent platelet aggregation	(73)
Circular 3D stenosis channel	3D printing; Soft lithography	Pump-driven constant flow input and geometry mediated flow output	HUVECs	Citrated human WB	Flow-mediated blood-EC interaction and thrombosis	(56)
Flexible micropost array	Soft lithography	No external mechanical input	FNG or FNT coated array	Platelet-rich plasma	Platelet contractile forces characterization	(49)
**Two-layered microfluidics**						
Parallel channels with non-uniform heights and widths	Soft lithography	Gravity driven constant flow input and geometry mediated flow output	ECM molecules coated bare channel	Human/mouse WB	Shear-dependent platelet adhesion	(66)
Microscale blocks and flexible posts in the channel	Assembly soft lithography	Pump-driven constant flow input and geometry mediated flow output	CGN or VWF coated bare channel	Citrated human WB	Hemodynamic effect on platelet contractility	(40)
**Three-layered microfluidics**						
Assembled web network	Assembly soft lithography; Injection molding with Type 1 Collagen hydrogel	Gravity driven constant flow input and geometry mediated flow pattern	HUVECs only or cocultured with HBVPCs/HUASMCs	Citrated human WB	Angiogenesis and thrombosis; Flow-driven assembly of VWF fibers and its interaction with platelet	(67)
Two chambers separated by porous membrane layer	3D printing; Assembly soft lithography	Pump-driven constant flow input and output	HUVECs/HPMECs cocultured with alveolar epithelium	Recalcified human WB	Platelet-endothelial dynamics in pulmonary thrombosis	(61)
Top pressure actuation chamber, middle diaphragm, bottom channel	Assembly soft lithography; injection molding	Pneumatic peristaltic pump-driven flow	Type 1 CGN coated bare channel	Citrated human WB/washed platelet	Platelet thrombosis assay and antiplatelet drug screening	(55)
Micropatterns printed on microfluidic channel	Assembly soft lithography; micropattern with stamping; hydrogel casting	Pump-driven constant flow and ECM mediated stiffness stimuli	FNG coated micropattern	Platelet-rich plasma	Hemodynamic and ECM influences on platelet contractility	(68)

*WB, Whole Blood; EC, Endothelial cell; HUVECs, Human umbilical vein endothelial cells; HMVECs, Primary human cardiac microvascular endothelial cells; HBVPCs, Human brain vascular pericytes; HUASMCs, Human umbilical arterial smooth muscle cells; HLMVECs, Human lung microvascular endothelial cells; HPMECs, Primary Human Pulmonary Microvascular Endothelial Cells; ECM, Extracellular matrix; TNF-α, tumor necrosis factor-alpha; VWF, von Willebrand Factor; TF, Tissue factor; FNG, Fibrinogen; CGN, Collagen*.

### Shear Dependent Platelet Adhesion

Earlier endeavors in the field focused on developing high-throughput microfluidic systems with parallel straight microchannels to assess shear effects on platelet thrombotic functions (21). One of the novel straight channel microfluidic models was developed by Gutierrez et al., that validated the use of adhesion assays with VWF, fibrinogen, or collagen-coated substrates (66). The model contained eight rectilinear channels that covered a 100-fold range of shear rates to recapitulate the venous and arterial flow conditions. Such approaches can achieve high-throughput examination and avoid matrix variability between different microchannels. Similar studies were later conducted by Conant et al., who developed a 2D narrowing microfluidic device consisting of 24 microchannels coated with a range of matrix proteins on a 48-well plate. Each of their microchannels was coupled with two wells (Figure 1A) (70). By changing the flow inputs with a syringe pump, platelet adhesion assays were conducted under controllable physiological and pathological hemodynamic conditions. These two high-throughput microfluidic systems substantially shortened the time for platelet adhesion analysis. As a key advantage, these microdevices use small volume of blood samples to produce a range of replicable results.

### Flow Disturbance Dependent Platelet Aggregation

In pathological contexts, the aforementioned straight microfluidics could not effectively recapitulate flow disturbance at stenotic and bifurcated vasculatures (74).

To this end, Tovar-Lopez et al. proposed a 2D stenosed microfluidic model to assess the role of shear gradients on platelet aggregation at three observation zones: the pre-stenosis for shear acceleration; the apex of stenosis for maximum shear; and the post-stenosis for shear deceleration (46). Interestingly, stable platelet aggregation was only found at the post-stenotic zone with shear deceleration. Costa et al. presented similar observation in 3D coronary artery mimicking microfluidics. The 3D vessel geometries and stenoses were reconstructed from computed tomography angiography, and then manufactured into a microfluidic channel using stereolithography 3D-printing and PDMS soft lithography (56). The computational fluid dynamic simulation identified that backflow at the distal stenosis associates with rapid platelet aggregation. These results were comparable to the studies by Menon et al., using circular microfluidics with various degrees of stenosis in 2D and 3D (57). When Vascularized with endothelial cells, these microfluidics revealed that elevated shear stress at the stenotic regions activated endothelial cells into pro-thrombotic and pro-inflammatory states. Nevertheless, the round surface of these circular microfluidics makes it difficult in visualizing platelet adhesion and aggregation.

In addition, new microfluidic designs were made to investigate the prothrombotic role of shear gradients with respect to vessel bifurcations. The bifurcations produce flow separations, shear gradients, and oscillatory flows which promote atherosclerotic lesion initiation and thrombus formation (75). Li et al. designed a bifurcated stenosis device to mimic constricted regions in coronary arteries, where a mother microchannel is subdivided into two smaller daughter microchannels (73). Occlusive thrombi were formed extensively at higher shear rates between 1,500 and 4,000 s^−1^, but not at lower shear rates <1,500 s^−1^. Additionally, the complex hemodynamics at vessel bifurcations were also investigated in a multi-bypass ladder model developed by Zilberman-Rudenko et al. which consisted of two parallel channels and 10 T-junction-like bypasses (47). Thrombus formation was found to be heightened at channel intersections which displaying skewed flow profiles, stagnation zones, flow separation, recirculation zones, and shear gradients.

Furthermore, research has been undertaken into automating the occlusion time calculation as thrombi form. Jain et al. designed a stenosed arteriole mimicking microfluidic device with multiple regions of pre- and post-stenosis (Figure 1C) (71). A mathematical model was used to quantitate shear gradient induced thrombosis in real time, indicating the steady reaction, growth, and saturation phases of thrombus development. They have also demonstrated the use of this microdevice to assess antithrombotic drug effects on individual patients.

### Platelet Contraction

Activated platelets in newly formed aggregates are highly contractile with respect to thrombus stabilization and consolidation (76–81). Recent studies have sought to analyze the degree of forceful cytoskeletal contractions in activated platelets. Notably, Muthard and Diamond designed a microblock microfluidic model composed of a primary flow channel and a side scaffold channel to investigate platelet contractile forces during thrombus formation under arterial shear conditions (77). Platelet contraction occurred dramatically during flow arrest, and a consequent increase in the clot permeability was observed (77, 78). While Liang et al. quantified platelet retraction force without physiological flow using flexible post array (49), Ting et al. devised a microfluidic arrays of rectangular blocks and flexible microposts which were subjected to arterial blood flow (Figure 1D) (40, 79). They measured the deflections of microposts caused by contracting platelet aggregates around the rectangular block, thereby quantifying the ensemble platelet contractile forces, in which myosin II, ADP activation and integrin GPIIb/IIIa were found to play key roles. Further, Myers et al. designed an array of fibrinogen-coated microdots patterned on a hydrogel substrate, where platelets adhering to the microdot extended filopodia to the adjacent microdot (68). The displacement of the microdots due to platelet contraction enabled quantification of platelet contractility in a cytometry manner. Evidently, the micropost array is advantageous to visualize and measure platelet contractility and can be extended to the diagnosis of reduced platelet contractility associated with bleeding conditions including Wiskott Aldrich Syndrome (WAS) (68) and Immune Thrombocytopenia (ITP) (82).

### Platelet–VWF Association

In addition to the hemodynamic microenvironment, platelets interact with the circulatory components including the vessel walls, blood cells (red blood cells, T-cells) and plasma proteins (VWF, fibrinogen, tissue factor, collagen) (83). Particularly, VWF is central to primary hemostasis in mediating platelet recruitment to the damaged vascular subendothelium and subsequent platelet aggregation (84). The critical role of VWF in regulating platelet biomechanical adhesion and aggregation was investigated in numerous microfluidic systems.

Notably, Dong et al. employed a commercial parallel plate flow chamber comprising a polycarbonate slab, silicon gasket and an endothelial cell-lined glass coverslip to study platelet GPIbα–VWF mediated platelet adhesion (85). Upon endothelial activation, platelets adhered to the released ultra-large VWF and formed extraordinary long ‘beads-on-a-string'-like structure at a venous shear rate. Using the same parallel plate device, Padilla et al. proposed to reduce platelet-endothelial VWF interaction by exposing the VWF-A2 domain to ADAMTS13 under elongational shear (86). However, such parallel plate flow chamber cannot investigate more disturbed hemodynamic conditions due to its laminar flow profile.

Zheng et al. designed a complex endothelialized microvascular network (Figure 1E) (67). Such microfluidic model was fabricated by injection molding type I collagen between two plexiglass pieces and PDMS for stabilization. Zheng et al. then optimized the model to incorporate multiple vascular geometries including straight, grid, tortuous, stenosed channels (15) to investigate flow pattern-driven platelet–VWF interaction. The fluid dynamic profile near the bifurcation and vessel junctions enhanced VWF self-association, causing irreversible rapid platelet adhesion and aggregation. Westein et al. fabricated an atherosclerotic vessel mimicking microfluidic device with a half-circular eccentric stenosis (20–80% stenosis) and confirmed that plasma VWF strongly influenced the proaggregatory response (Figure 1F) (6). The VWF multimers were found to elongate with shear gradients and occupied a 15-times higher surface area in deceleration zones promoting extensive platelet aggregation. The significance of VWF was further supported by Kim et al. using a stenotic microfluidic model (87). As such, complex platforms with diverse geometries is beneficial to the study of thrombotic therapies targeting VWF self-association and the subsequent platelet responses (88).

### Platelet–Endothelium Interaction

Endothelial expression of P-selectin, E-selectin, vascular cell adhesion molecule 1 (VCAM-1) and intercellular adhesion molecule 1 (ICAM-1) can regulate platelet activation and adhesion (83). Understanding the influence of the endothelium on platelet binding kinetics is essential to unravel the mechanism underlying platelet thrombus formation.

Ciciliano et al. employed a T-shape endothelialized microfluidic platform to recapitulate FeCl_3_-induced thrombosis *in vitro* (72). The platform is composed of a main channel where endothelial cells were cultured to form a confluent endothelium, and a side channel where FeCl_3_ was infused to join the main channel to create the blood-FeCl_3_ interface. The FeCl_3_ influx induced endothelial injury, followed by the binding between Fe^3+^ ions and negatively charged proteins, which lead to platelet activation and occlusive aggregation. Dupuy et al. developed a thromboinflammation model by using six parallel microchannels with fixed human umbilical vein endothelial cells (HUVECs) alignment (16). Tumor necrosis factor alpha (TNF-α) treatment stimulated the expression of ICAM-1 and VCAM-1, and the secretion of VWF and tissue factor on the endothelium, leading to a dose-dependent increase in platelet–endothelial binding. Further, Jain et al. utilized the same model and found that TNF-α activated HUVECs increased E-selectin expression (41).

Notably, Jain et al. exhibited a triple-layered human lung alveolus-on-chip to study the interplay between platelets, vessel wall, and blood flow (Figure 1G) (61). The model mainly contains an upper microchannel coated with primary human lung alveolar epithelial cells and a lower microchannel lined with HUVECs, separated by a thin and porous membrane. Remarkably, TNF-α and lipopolysaccharide endotoxin stimulated the alveolar epithelium and increased the pulmonary vascular permeability, resulting in enhanced ICAM-1 expression and platelet-endothelial binding.

## Microfluidic Standardization

Increased usages of shear-based microdevices by different laboratories for examining platelet functions lead to heterogenous device characteristics. Lack of standardization in microfluidic designs and operating methods complicates the result comparison and reproducibility, which impedes translation into both clinical practices and industrial settings (89, 90). The ISTH Biorheology subcommittee has advocated for standardization of flow chamber-based thrombus formation assays (34, 89, 91, 92). Requirements for microfluidic standardization can be extended to the following considerations.

(i) Structural design. To test the abnormalities in platelet mechanosensing, several microfluidic designs can be standardized: shear dependent platelet aggregation can be assessed in straight channels; shear gradient dependent thrombus development can be investigated in stenotic channels; and micropillar array can be standardized for platelet contractility measurements (5). Standardizations of these geometries and dimensions ensure unified microfluidics to be used for platelet function tests by different laboratories (93).
(ii) Surface treatment. Adhesive ligands should be considered carefully to induce platelet adhesion, aggregation and contraction (94–97). Specifically, concentration, duration of treatment, molecular composition, coating procedure of the adhesive proteins needs to be specified for standard usage (95). Moreover, the source, type, passage number, culture medium, seeding procedure, culture duration, inflammatory stimulation of cell-derived surface should be considered thoroughly.
(iii) Sample preparation and perfusion. It is important to ensure appropriate handling of the perfusion samples which include whole blood and washed platelets. Source of blood samples, procedure and handling of blood collection, storage, and the use of anticoagulants are to be standardized (94).
(iv) Hemodynamic settings. Microfluidic device materials, tubing dimensions, perfusion pumps and choice of bulk shear rates are essential when conducting flow tests (98). Standardizations of these hemodynamic settings including the inertial forces and Reynolds number within the microfluidic systems will allow more certain conclusions to be extrapolated (93).
(v) Visualization and quantification methods. Standardization of microscopy-based analytical approaches can be based on different stages of thrombus formation indicated by platelet adhesion receptors, activated ligand expression and aggregate volume. The types, sources, and concentrations of fluorescent stains and antibodies should be specified to control adverse chemical and phototoxic effect on platelet functions. The method of quantification of the fluorescent microscopy data needs to be standardized to allow more transparency in obtained results.

## Commercially Available Microfluidics for Laboratory Use

The geometry of microfluidic devices is of the utmost relevance to their application in platelet function studies and their extension to antiplatelet drug screening platforms. Commercially available microfluidic systems have been able to standardize more simple, quasi-2D planar geometries but are still limited by their inability to create complex geometry in a high-throughput manner. A summary of studies looking at platelet biology in thrombosis using commercialized microfluidic slides is presented in Table 2.

**Table 2:** Commercialized microfluidic slides for laboratory research.

**Flow chamber**	**Surface treatment**	**Vasculature status**	**Shear rate(dyne/cm^**2**^)**	**Perfusion sample**	**Investigation**	**References**	**Representative image**
Ibidi μ-slide VI^0.4^	Collagen /thrombin	No ECs	Static	Washed platelet	Platelet activation plasminogen activator inhibitor 1	(99)	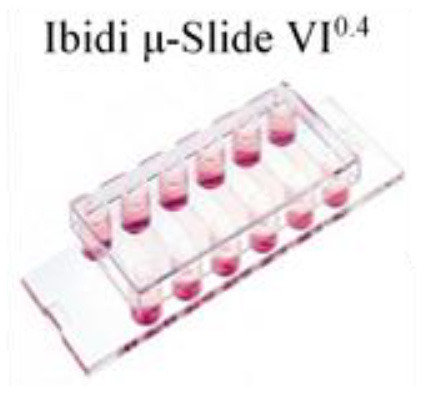
Ibidi μ-slide VI^0.4^	No specific coating	HPAECs	150 s^−1^	Mouse/human WB	Platelet activation lethal sepsis	(100)	
Ibidi μ-slide VI^0.4^	Human Fc-podoplanin fusion protein	LECs /HUVECs	50–1,350 s^−1^	Mouse/human WB	Effect of CLEC-2-podoplanin interactions on Platelet adhesion	(101)	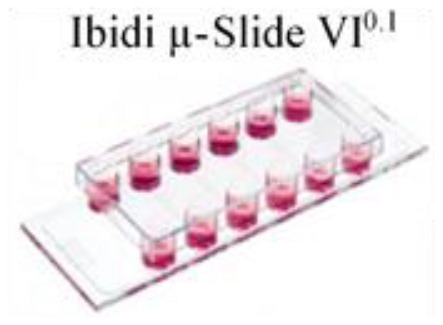
Ibidi^Ò^ μ-slides	Fibrinogen	No ECs	Static	Human WB	Platelet adhesion and thrombo-inflammation	(102)	
Ibidi^Ò^ μ-slides/Biotechs FCS2 flow chamber	Collagen type 1	No ECs	200–1,000 s^−1^	Washed platelets	Platelet–monocyte interactions	(103)	
Cellix Vena8 Fluoro+ biochip	Fibrillar collagen	No ECs	200–1,000 s^−1^	Washed platelet/Human WB	Effects of β amyloid peptides on platelet	(104)	
Cellix Vena8 Fluoro + biochip	Collagen type 1	No ECs	90 s^−1^	Platelet-rich plasma	Effect of omega-3 fatty acids on platelet aggregation	(105)	
							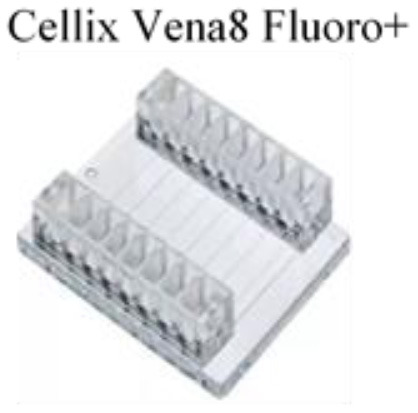
Cellix Vena8 biochips	Collagen/fibrinogen	No ECs	0.5 dyne/cm^2^	Platelet-rich plasma/human WB	Effect of prostaglandin on platelet aggregation	(106)	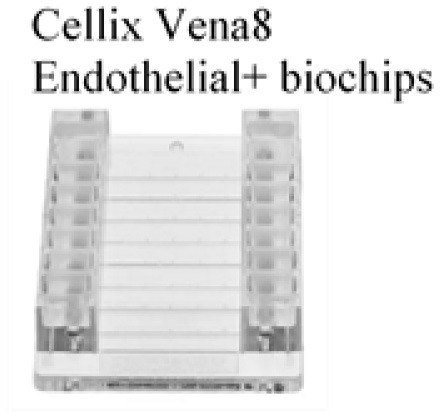
Cellix Vena 8 Endothelial + biochips	Fibronectin	HCAECs	0.5 dyne/cm^2^	Washed platelets	Effect of plasma protein on platelet-endothelium crosstalk	(107)	
Cellix Vena8 Fluoro+ biochip	Collagen type 1	No ECs	67.5 dyne/cm^2^	Human WB	Effect of Gut Microbial Metabolite TMAO on platelet and thrombosis	(99)	
Cellix Vena8 biochips	Fibrinogen/collagen	HUVECs w/wo TNF-α stimulation	0.3 dyne/cm^2^	Unwashed platelet-rich plasma/washed platelet	Platelet activation and adhesion to diseased endothelium	(100)	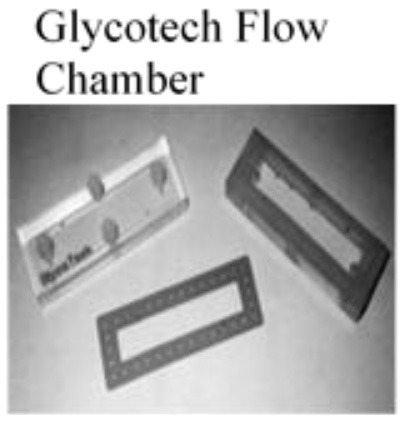
Cellix Vena8 Fluoro+ biochip	Equine tendon collagen	No ECs	10 dyne/cm^2^	Platelet-rich plasma	Effect of legacy perfluoroalkyl substances on platelet dynamics	(101)	
Glycotech parallel-plate flow chamber	No specific coating	HUVECs/HUAECs/HMVEC	2–50 dyne/cm^2^	Washed platelets	VWF- platelet interaction in TTP	(102)	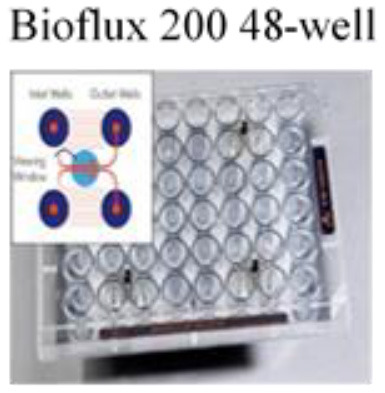
Glycotech Parallel-plate flow chamber	No specific coating	HUVECs	2.5 dyne/cm^2^	Washed platelets	VWF- platelet interaction	(103)	
Fluxion Biosciences, high-shear, 48-well Bioflux plates	Fibronectin	HUVECs w/wo TNF-α stimulation	5 dyne/cm^2^	Human WB	TF-driven platelet adhesion and aggregation	(104)	
Fluxion Biosciences, 48-well Bioflux plates	Fibronectin	HUVECs	10 dyne/cm^2^	Platelet-rich plasma/human WB	PF4-heparin interaction in HIT	(105)	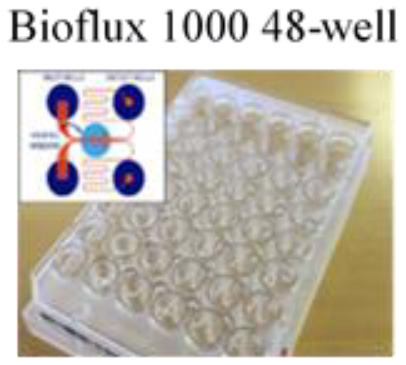
Fluxion Biosciences, 48-well Bioflux plates	Fibronectin	HUVECs	10 dyne/cm^2^	Human WB	PF4-VWF-HIT antibody interaction	(106)	

*WB, Whole Blood; ECs, Endothelial cells; HUVECs, Human umbilical vein endothelial cells; LECs, Lymphatic endothelial cells; HPAECs, Human Pulmonary Artery Endothelial Cells; HUAECs, Human Umbilical Artery Endothelial Cells; HMVECs, Primary human cardiac microvascular endothelial cells; TNF-α, tumor necrosis factor-alpha; TMAO, Trimethylamine N-oxide; VWF, von Willebrand Factor; TF, Tissue factor; HIT, Heparin-induced thrombocytopenia; PF4, platelet factor 4; CLEC-2, C-type lectin-like type II transmembrane receptor. HCAECs: Human Coronary Aortic Endothelial Cells*.

For effective commercialization of more complicated microfluidic structures, the fabrication protocol need to be high-throughput, robust, reproducible and cost-efficient (108). Such fabrication techniques including nanoimprint lithography, 3D printing and anodic aluminum oxidation are promising in supporting rapid-prototyping of highly tailorable complex microfluidics with replicable specifications (90, 108). With increased standardization and advancements in the commercial capacity, biomimetic microfluidic systems will accelerate novel antiplatelet therapeutic development and have great potential in patient screening (109).

## Conclusion

Emerging microfluidic technologies have advanced our understanding of platelet mechanobiology and its role in hemostasis and thrombosis. The ease of manufacturing complex geometries, versatility and the high throughput of microfluidic experiments have enabled researchers to investigate how platelets respond to their hemodynamic microenvironment and interact with the circulatory system in a well-controlled biomechanical milieu. The varieties in designs, fabrications, operation procedures and analytical approaches of customized microfluidics have urged the standardization need for reproducibility. The increasing use of commercially available microfluidics accelerates the translation of platelet mechanobiology to pre-clinical and industrial applications. In the next decade, we foresee microfluidic technologies to be used for patient-specific disease management, diagnosis, as well as antiplatelet drug screening.

## Author Contributions

YZ and LJ conceived the study and wrote the manuscript. VC, RP, and SR co-wrote the manuscript. HL and LJ provided critical comments, suggestions, and text. LJ designed and supervised the study. All authors contributed to the article and approved the submitted version.

## Funding

This work was supported by the Australian Research Council (ARC) Discovery Project (DP200101970 - LJ), the National Health and Medical Research Council (NHMRC) of Australia Ideas Grant (APP2003904 – LJ), NSW Cardiovascular Capacity Building Program (Early-Mid Career Researcher Grant - LJ), Sydney Research Accelerator prize (SOAR - LJ), and Ramaciotti Foundations Health Investment Grant (2020HIG76 - LJ). LJ is an ARC DECRA fellow (DE190100609) and National Heart Foundation Future Leader fellow (FLF2 105863).

## Conflict of Interest

The authors declare that the research was conducted in the absence of any commercial or financial relationships that could be construed as a potential conflict of interest.

## Publisher's Note

All claims expressed in this article are solely those of the authors and do not necessarily represent those of their affiliated organizations, or those of the publisher, the editors and the reviewers. Any product that may be evaluated in this article, or claim that may be made by its manufacturer, is not guaranteed or endorsed by the publisher.
